# Reduced menin expression leads to decreased ERα expression and is correlated with the occurrence of human luminal B-like and ER-negative breast cancer subtypes

**DOI:** 10.1007/s10549-021-06339-9

**Published:** 2021-09-24

**Authors:** Romain Teinturier, Razan Abou Ziki, Loay Kassem, Yakun Luo, Lucie Malbeteau, Samuele Gherardi, Laura Corbo, Philippe Bertolino, Thomas Bachelot, Isabelle Treilleux, Chang Xian Zhang, Muriel Le Romancer

**Affiliations:** 1 Univ Lyon, Université Claude Bernard Lyon 1, INSERM 1052, CNRS 5286, Centre Léon Bérard, Centre de Recherche en Cancérologie de Lyon, 69008 Lyon, France; 2 Clinical Oncology Department, Faculty of Medicine, Cairo University, Cairo, Egypt; 3 Department of Medical Oncology, Centre Léon Bérard, Lyon, France; 4 Département de Biopathologie, Centre Léon Bérard, 69008 Lyon, France

**Keywords:** Menin, *ESR1*, ERα, GATA3, Breast cancer, Luminal subtypes

## Abstract

**Purpose:**

Menin, encoded by the *MEN1* gene, was recently reported to be involved in breast cancers, though the underlying mechanisms remain elusive. In the current study, we sought to further determine its role in mammary cells.

**Methods:**

Menin expression in mammary lesions from mammary-specific *Men1* mutant mice was detected using immunofluorescence staining. RT-qPCR and western blot were performed to determine the role of menin in ERα expression in human breast cancer cell lines. ChIP-qPCR and reporter gene assays were carried out to dissect the action of menin on the proximal *ESR1* promoter. Menin expression in female patients with breast cancer was analyzed and its correlation with breast cancer subtypes was investigated.

**Results:**

Immunofluorescence staining revealed that early mammary neoplasia in *Men1* mutant mice displayed weak ERα expression. Furthermore, *MEN1* silencing led to both reduced *ESR1* mRNA and ERα protein expression in MCF7 and T47D cells. To further dissect the regulation of *ESR1* transcription by menin, we examined whether and in which way menin could regulate the proximal *ESR1* promoter, which has not been fully explored. Using ChIP analysis and reporter gene assays covering − 2500 bp to + 2000 bp of the TSS position, we showed that the activity of the proximal *ESR1* promoter was markedly reduced upon menin downregulation independently of H3K4me3 status. Importantly, by analyzing the expression of menin in 354 human breast cancers, we found that a lower expression was associated with ER-negative breast cancer (*P* = 0.041). Moreover, among the 294 ER-positive breast cancer samples, reduced menin expression was not only associated with larger tumors (*P* = 0.01) and higher SBR grades (*P* = 0.005) but also with the luminal B-like breast cancer subtype (*P* = 0.006). Consistent with our clinical data, we demonstrated that GATA3 and FOXA1, co-factors in *ESR1* regulation, interact physically with menin in MCF7 cells, and *MEN1* knockdown led to altered protein expression of GATA3, the latter being a known marker of the luminal A subtype, in MCF7 cells.

**Conclusion:**

Taken together, our data provide clues to the important role of menin in ERα regulation and the formation of breast cancer subtypes.

**Supplementary Information:**

The online version contains supplementary material available at 10.1007/s10549-021-06339-9.

## Introduction

Breast cancers are among the most common malignancies worldwide and remain the leading cause of cancer-related mortality in women [1]. Previous receptor expression analyses enabled their classification into 4 major clinical subtypes, including luminal A, luminal B, HER2-enriched and triple-negative [2]. The luminal A subtype encompasses approximately 44% of breast cancers. This subtype is estrogen receptor (ER)-positive and/or progesterone receptor (PR)-positive and human epidermal growth factor receptor 2 (HER2)-negative, which displays a reduced expression of proliferation-related genes [3] and is sensitive to endocrine therapy with an overall favorable prognosis. The luminal B subtype represents around 20% of breast cancers and displays lower expression of ERα-related genes, a variable expression of (HER2), and a higher expression of proliferation-related genes [4]. This subtype harbors more genomic instability and has a poorer prognosis than the luminal A subtype [5]. The HER2-enriched subtype is ER-negative, PR-negative, HER2-positive, and highly sensitive to therapies targeting the HER2 receptor. The triple-negative breast cancer (TNBC) subtype is negative for all three receptors [6] and is the most aggressive with the worst prognosis.

Patients harboring *MEN1* mutations are predisposed to multiple endocrine neoplasia type 1 (MEN1) syndrome, which is associated with multi-occurring endocrine tumors [7], as well as several types of non-endocrine tumors [8]. Numerous studies have revealed that menin is a multifaceted protein involved not only in the development and control of cell growth of endocrine cells but also in a variety of biological processes, including hematopoiesis and osteogenesis [9–11]. The wide range of biological functions regulated by menin results from its interaction with numerous proteins [12]. These menin-interacting proteins include transcription factors (the components of AP1, NFkB, and the TGFβ signaling pathways) and chromatin-modifying proteins (mixed lineage leukemia (MLL), Sin3A, and HDAC) [12, 13]. Notably, menin physically interacts with a range of nuclear receptors, including ERα and the androgen receptor (AR), to regulate their pathways [14–16].

Over the last few years, evidence has emerged, in vivo, to suggest that menin may play a role in breast cancers [17]. (1) Female heterozygous *Men1* knockout mice develop cancers of mammary cells with a low frequency [18], and conditional mammary gland-specific *Men1* disruption leads to the development of mammary intraepithelial neoplasia (MIN) in over 50% of female mutant mice [19]. (2) Importantly, an exhaustive analysis of several cohorts of MEN1 patients revealed a significant predisposition to breast cancer [20]. (3) Menin downregulation was detected in a substantial proportion of human sporadic breast cancer samples [19], and *MEN1* mutations were found, although rarely in sporadic breast cancers, justifying its addition to the list of driver mutations/genes of this pathology [21, 22]. Of note, the abovementioned analyses all highlight the suppressive role of menin in mammary cell tumorigenesis. However, Imachi et al*.* found that, among 65 ERα + breast cancer samples treated with tamoxifen, menin-positive tumors (20 patients) had worse clinical outcome and were more resistant to tamoxifen than menin-negative tumors, suggesting that menin exerts oncogenic effects in these cases [15]. Interestingly, a recent publication, revealing the role of menin in regulating the enhancer of the *ESR1* gene coding for ERα, suggests distinct functions for menin in primary normal mammary cells and in breast cancers [23]. The authors showed that, although menin possesses a crucial tumor-suppressive role in normal mammary cells, it acts as an oncogenic factor in ERα + breast cancer cell lines through an enhancer-mediated regulation of *ESR1* transcription. Notably, they demonstrated, by the ZR75-1 breast cancer cell line which does not express menin, that the re-expression of menin leads to enhanced ERα expression. Given the heterogeneous nature of breast cancers, we sought to further investigate the regulation of *ESR1* by menin and assess the putative relationship between *ESR1* dysregulation due to menin inactivation and the occurrence of human breast cancer subtypes.

### Results

### *Men1* deficiency in mice leads to ERα downregulation in early mammary lesions

**Fig. 1:**
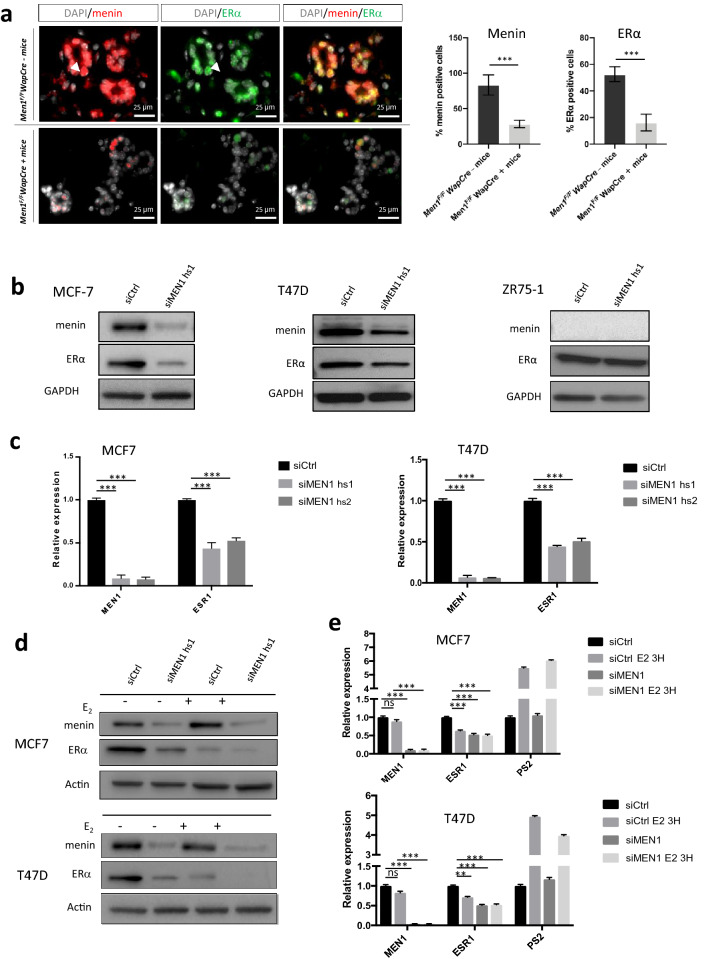
Reduced menin expression leads to a decrease in ERα expression. **a** Co-immunofluorescence against menin and ERα on mammary gland sections from *Men1*^*F/F*^*WapCre*^*−*^ mice (upper panel) and *Men1*^*F/F*^*WapCre*^+^ mice (lower panel) at < 12 months of age. Quantification of IF signals for menin and ERα is shown on the right*.* **b** Western blot analyses using antibodies against menin and ERα in MCF7, T47D, and ZR75-1 cells treated with siRNA control (siCtrl) or siRNA targeting the *MEN1* gene (siMEN1 hs1). **c** Quantitative RT-qPCR analyses of *MEN1* and *ESR1* expression in MCF7 and T47D cells treated with siCtrl or two different siMEN1 (hs1 or hs2). **d** Western blot analyses using antibodies against menin and ERα in MCF7 and T47D treated with siRNA control (siCtrl) or siMEN1 hs1 and then subjected to estradiol (*E*_2_) stimulation at a concentration of 10 nM. **e** Quantitative RT-qPCR analyses of the *MEN1* and *ESR1* expression in MCF7 and T47D cells treated with siCtrl or siMEN1 hs1 and then subjected to estradiol (*E*_2_) stimulation at a concentration of 10 nM. *PS2* transcript was used as a positive control. All data are expressed as mean ± SEM, ns *P* > 0.05, *P < 0.05, **P < 0.01, ***P < 0.001

We previously reported the occurrence at a high incidence of mammary intraepithelial neoplasia (MIN) lesions, displaying weak ERα expression, in *Men1* mammary conditional mutant mice [19]. To further determine the causative role of menin deficiency in reduced ERα expression, we carried out double IF analysis of menin and ERα expression in normal and young mutant mice with MIN lesions, before the development of breast cancer. Three mice per control or mutant group were analyzed. As shown in Fig. 1a, all of the mammary luminal cells in *Men1*^*F/F*^*-WapCre*^*−*^ control mice expressed menin. Conversely, menin expression was lost in 71.1% of mammary cells in these young *Men1*^*F/F*^*-WapCre*^+^ mice (Fig. 1a). ERα was expressed in approximately 52.5% of luminal cells expressing menin in the former group (Fig. 1a, upper panel), whereas immunofluorescence revealed that ERα expression was nearly 3.2-fold lower in *Men1*-deficient cells in *Men1*^*F/F*^*-WapCre*^+^ mice (lower panel), compared to *Men1*^*F/F*^*-WapCre*^*−*^ mice. The merged images of menin and ERα staining clearly highlight that ERα is less expressed specifically in the nuclei of menin-deficient luminal cells (Fig. 1a).

### Menin downregulation in human ERα + mammary cells leads to reduced ERα expression

Next, we further dissected the regulation of ERα expression by menin using different approaches in ERα + breast cancer cell lines. To achieve this, we first performed *MEN1* knockdown (KD) using a siRNA approach. As shown in Fig. 1b, *MEN1* KD MCF7 and T47D cells displayed reduced ERα protein expression by western blot analysis, unlike the menin-negative ERα + cell line, ZR75-1. Moreover, *MEN1* KD led to a twofold decrease in *ESR1* mRNA levels by RT-qPCR analysis in MCF7 and T47D cell lines (Fig. 1c). We then verified the effects of *MEN1* KD on *ESR1* mRNA and ERα expression levels under estrogen (E_2_) stimulation. Western blot and RT-qPCR analyses showed that *MEN1* silencing further abrogated ERα expression under E_2_ stimulation but had no additional effect on *ESR1* transcription (Fig. 1d, e), most likely due to the fact that transcription had already reached its lowest level upon estrogen stimulation. All the above data thus confirm our in vivo analyses, indicating that menin is essential in maintaining *ESR1* transcription and ERα expression.

### Menin binds to the proximal region of the *ESR1* promoter

**Fig. 2:**
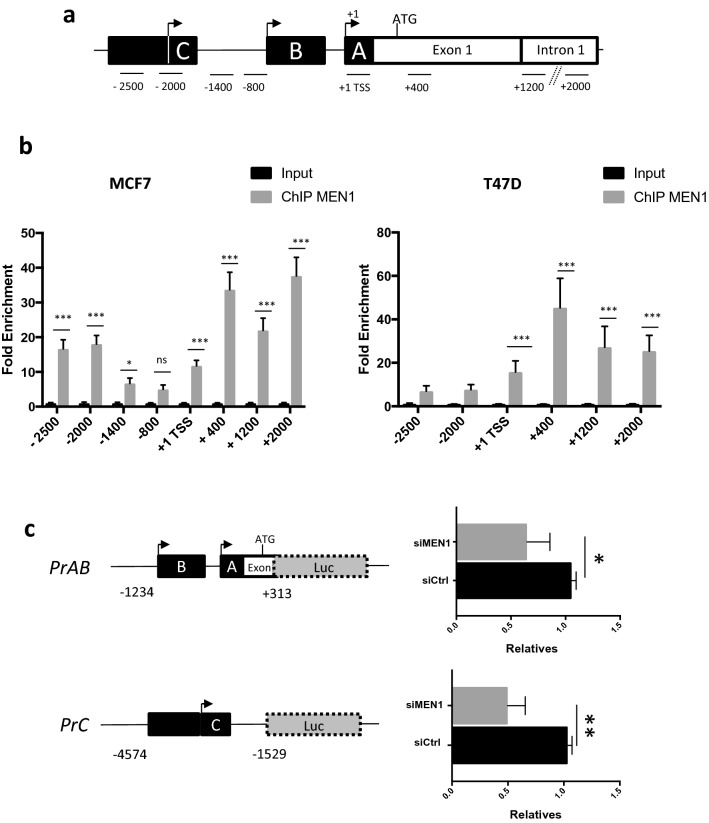
Recruitment and effects of menin on the proximal *ESR1* promoter. **a** Schematic diagram of the different regions of the *ESR1* promoter studied, including A, B, and C regions of *ESR1,* that were cloned into luciferase reporter constructs (see **c**). **b** ChIP-Quantitative PCR analyses of menin binding on the − 2500 bp/ + 2000 bp area flanking the transcription start site (TSS) of *ESR1* in MCF7 (left) and T47D (right) cells. **c** MCF7 cells were transfected with siCtrl or siMEN1 hs1 and pGL3-PrAB or pGL3-PrC luciferase reporter. After 48 h of transfection, luciferase activities were measured in triplicate for each condition. All the data are expressed as mean ± SEM, ns *P* > 0.05, *P < 0.05, **P < 0.01, ***P < 0.001

Dreijerink et al*.* reported that menin plays a crucial role in the regulation of *ESR1* transcription in an enhancer-mediated way [23]. We noticed that, although the study revealed the binding of menin at the transcription start site (TSS) of the *ESR1* promoter, no further analyses were reported on this region. We thus carried out ChIP analyses to evaluate the binding of menin to the − 2500 bp to + 2000 bp region around the TSS, defined based on previously reported works (Fig. 2a) [23, 24], to fully decipher the regulation of *ESR1* transcription by menin at the proximal promoter region. Menin was significantly enriched in the *ESR1* promoter region encompassing the TSS to + 2000 bp in MCF7 (Fig. 2b, left panel) and T47D (Fig. 2b, right panel) cells and more specifically in the promoter area C in MCF7 cells. Importantly, we confirmed by luciferase reporter assays that the transcriptional activity of the proximal *ESR1* promoter region A/B and C was markedly reduced when *MEN1* was knocked down (Fig. 2c).

**Fig. 3:**
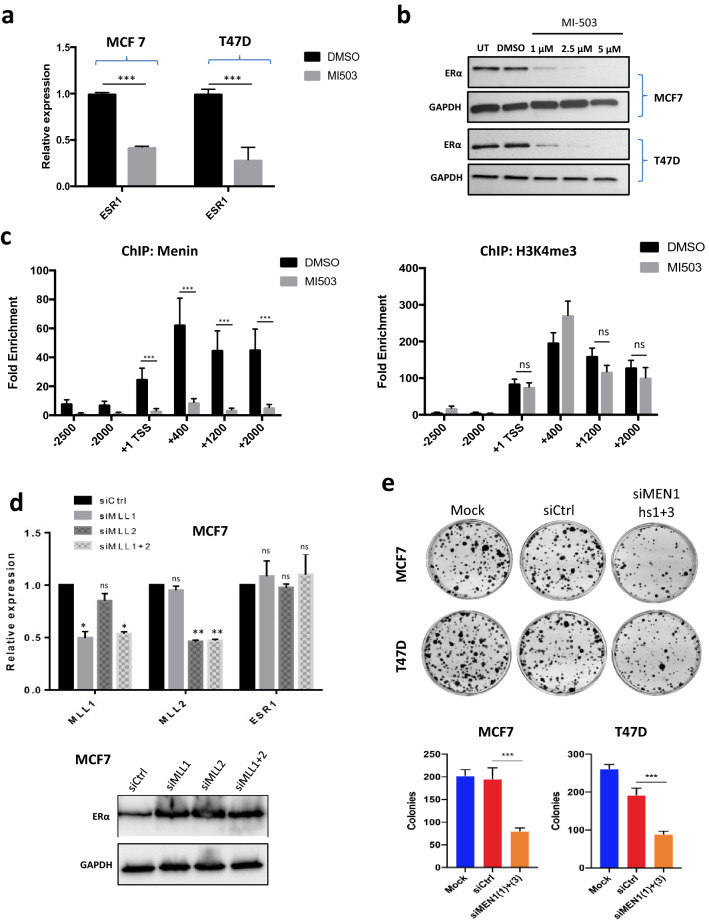
The regulation of the proximal *ESR1* promoter by menin does not entirely rely on the MLL complex. **a** Quantitative RT-qPCR analyses of *ESR1* expression in MCF7 and T47D cells treated or not with the inhibitor of menin–MLL interaction, MI503 at a concentration of 2 µM. **b** Western blot analyses of ERα expression in MCF7 and T47D cells untreated (UT) or treated with MI503 at concentrations of 1, 2.5, and 5 µM. **c** ChIP-qPCR analysis of the binding of the anti-menin antibody (left panel), or anti-H3K4me3 antibody (right panel) to the − 2500 bp/+ 2000 bp area flanking the transcription start site (TSS) on the *ESR1* in MCF7 cells treated or not with MI503 at a concentration of 2 µM for 48 h. **d** Quantitative RT-qPCR (left panel) and western blot (right panel) analyses of the expression of *MLL1* (*KMT2A*)*, MLL2* (*KMT2B*), and *ESR1* in MCF7 cells treated with siCtrl, siMLL1, siMLL2, or combined siMLL1 and siMLL2. **e** Representative images of foci formation assay with MCF7 and T47D cells treated with siMEN1(1) + (3) or siCrtl. Quantification of foci formation assay is shown on the right. All the data are expressed as mean ± SEM, ns *P* > 0.05, *P < 0.05, **P < 0.01, ***P < 0.001

On considering the data published by Dreijerink et al. [23] showing that H3K4me3 marks are more abundant in the proximal part of the *ESR1* promoter, we sought to investigate the involvement of the MLL complex, a major actor modifying H3K4me3 marks [13], in the regulation of the *ESR1* promoter. By treating MCF7 and T47D cells with an inhibitor of the menin–MLL interaction, MI503, RT-qPCR analyses unveiled a more than twofold decrease in *ESR1* transcription (Fig. 3a). Western blot analyses in MCF7 and T47D cells using the same inhibitor also revealed a decrease in ERα expression at the protein level (Fig. 3b). We then verified the potential alteration of H3K4me3 marks at this region upon inhibition of the MEN1/MLL complex. ChIP analysis with anti-H3K4me3 antibodies showed that, while MI503 treatment led to a markedly reduced binding of menin (Fig. 3c, left panel) at 48 h, H3K4me3 methylation was not altered upon menin/MLL inhibition in the tested region at this time point (Fig. 3c, right panel). We then performed the same analysis at 72 h and 96 h and found that at 72 h, one of the tested H3K4me3 marks significantly decreased, while other H3K4me3 marks slightly decreased but insignificantly (Fig. S1, upper panel). At 96 h, all H3K4me3 marks had decreased significantly, except for the one on the TSS site (Fig. S1, lower panel), whereas a substantial proportion of the MI503-treated cells stopped to grow. Furthermore, RT-qPCR analyses showed that neither siMLL1, nor siMLL2, nor their combination affected *ESR1* transcription (Fig. 3d, left panel) and ERα expression (Fig. 3d, right panel). Taken together, our data provide evidence that menin regulates the proximal *ESR1* promoter and raise the question of the involvement of factors other than the MLL complex in this regulation.

Having confirmed and extended the role of menin in regulating *ESR1* transcription, we sought to further confirm its role in the growth of ER+ breast cancer cells, as previously reported [23]. To achieve this, we used colony formation assays to investigate cell growth behavior after *MEN1* knockdown. As shown in Fig. 3e, *MEN1* silencing in both MCF7 and T47D cells led to reduced colony formation, supporting that menin is needed for the growth of these ER+ breast cancer cells.

### Lower menin expression is associated with luminal B-like and ER-negative breast cancer subtypes

Our observations prompted us to perform a thorough investigation of the levels of the menin protein in a cohort of breast cancer patients having undergone surgery at the Centre Léon Bérard (CLB) hospital from 2001 to 2003. Among 354 patients, 151 (42.7%) had a low menin expression, while 203 (57.3%) had a high expression. Among the 294 patients with ER+/HER2− tumors, 116 patients (39.5%) had a low nuclear menin expression and 178 patients (60.5%) had a high expression. In the cohort of 354 patients, we found that lower nuclear menin (*H* score ≤ 100) expression was significantly associated with ER-negative breast cancers (*P* = 0.041) and with the HER2-enriched subtype (*P* = 0.049, Table 1). Moreover, among the 294 ER+/HER2− patients, we observed that low menin expression was associated with the luminal B-like breast cancer subtype (*P* = 0.006), larger tumors (*P* = 0.016), and higher SBR grades (*P* = 0.005, Table 2).

**Table 1:** Correlation between menin expression and the clinico-pathological factors of 354 breast cancer patients

Variable	Menin low (≤ 100)		Menin high (> 100)		P*
	No. 151	(%) (42.7)	No. 203	(%) (57.3)	
Age (year)					
Mean (± SD)	59.2	(± 13.3)	58.6	(± 11.2)	0.081^†^
Age groups					
≤ 50 years	31	(20.5)	58	(28.6)	0.085
> 50 years	120	(79.5)	145	(71.4)	
BMI					
≤ 25	78	(53.4)	133	(67.9)	**0.007**
> 25	68	(46.6)	63	(32.1)	
T. size					
≤ 2 cm	72	(47.7)	131	(64.5)	
> 2 cm	79	(52.3)	72	(35.5)	**0.002**
LN invasion					
No	64	(42.4)	84	(41.1)	
Yes	87	(57.6)	119	(58.6)	0.85
SBR grade					
Gr 1	16	(10.6)	46	(22.7)	**0.001**
Gr 2	68	(45.0)	102	(50.2)	
Gr 3	67	(44.4)	55	(27.1)	
ER status					
Negative	26	(17.2)	20	(9.9)	**0.041**
Positive	125	(82.8)	183	(90.1)	
PR status					
Negative	44	(29.1)	44	(21.7)	0.108
Positive	107	(70.9)	159	(78.3)	
Her 2 status					
Negative	84	(89.6)	100	(95.2)	**0.049**
Over-expressed	12	(10.4)	5	(4.8)	

Bold indicates statistically significant values

*Correlation by Pearson’s *χ*^2^ test unless otherwise specified

^†^Difference between means analyzed using the Student’s *t* test

**Table 2:** Correlation between menin expression and the clinico-pathological factors of 294 ER^+^/HER2^−^ breast cancer patients

Variable	Menin low		Menin high		P*
	No. 116	(%) (39.5)	No. 178	(%) (60.5)	
Age groups					
≤ 50 years	21	(18.1)	51	(28.7)	**0.040**
> 50 years	95	(81.9)	127	(71.3)	
Menopausal status					
Premenopausal	22	(19.9)	56	(32.6)	0.053
Postmenopausal	92	(80.1)	118	(67.4)	
BMI					
≤ 25	64	(57.1)	116	(67.8)	**0.045**
> 25	48	(42.9)	55	(32.2)	
T. size					
≤ 2 cm	62	(53.4)	120	(67.4)	**0.016**
> 2 cm	54	(46.6)	58	(32.6)	
LN invasion					
No	46	(39.7)	74	(41.6)	0.744
Yes	70	(60.3)	104	(58.4)	
SBR grade					
Gr 1	16	(13.8)	46	(25.8)	**0.005**
Gr 2	62	(53.4)	99	(55.6)	
Gr 3	38	(32.8)	33	(18.5)	
PR status					
Negative	18	(15.5)	21	(11.8)	0.358
Positive	98	(84.5)	157	(88.2)	
Breast Ca. subtype					
Luminal A	67	(57.8)	130	(73.0)	**0.006**
Luminal B	49	(42.2)	48	(27.0)	
Type of adjuvant hormonal					
Tamoxifen	51	(45.5)	81	(45.8)	0.970
AI	61	(54.5)	96	(54.2)	
Type of adjuvant chemo					
Anthracyclin	51	(96.2)	77	(88.5)	0.509
Anthra/Taxane	2	(3.8)	9	(10.3)	
Other	0	(0)	1	(1.1)	

Bold indicates statistically significant values

*Correlation by Fisher’s exact test

**Fig. 4:**
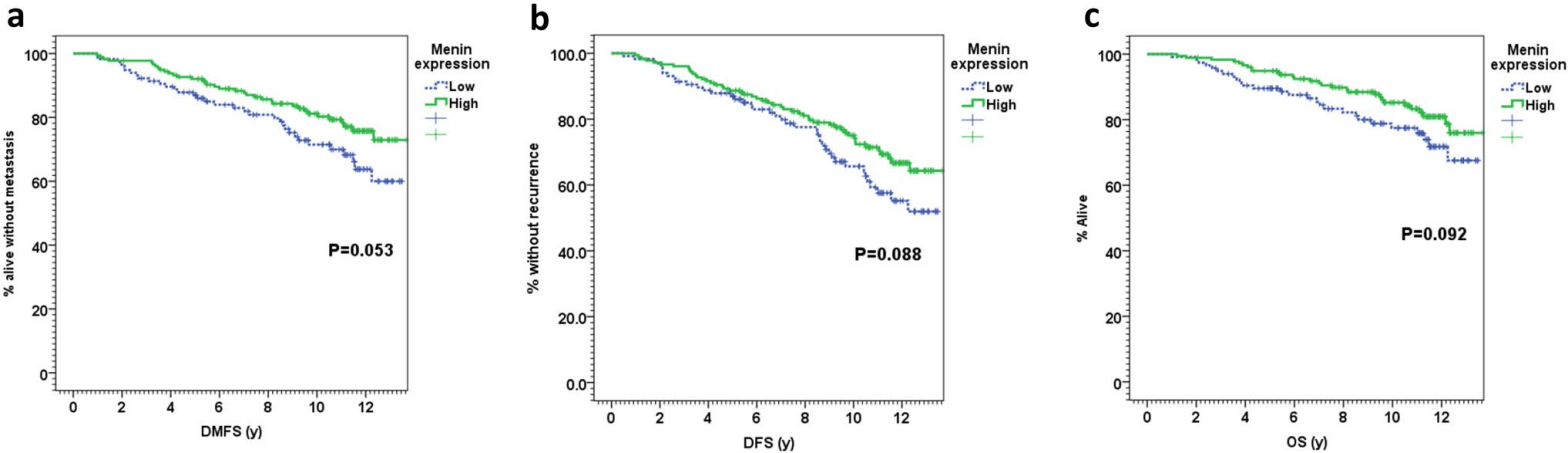
Comparison of Kaplan–Meier estimates for **a** distant metastasis-free survival (DMFS); **b** disease-free survival (DFS); and **c** overall survival (OS) in the ER^+^/HER2^−^ cohort of breast cancer patients included in the TMA of breast cancer patients, showing the survival curves of tumors with high (> 100) (green curve) or low menin nuclear staining (≤ 100) (blue curve)

Interestingly, among the ER+/HER2− cohort, we found that low menin expression was associated with worse distant metastasis-free survival (DMFS), with a 10-year DMFS of 71.5% *versus* 81.2% in patients with high menin expression, *P* = 0.053 (Fig. 4a). Furthermore, low menin expression was also associated with a trend for worse disease-free survival (DFS), with a DFS of 65.7% at 10 years *versus* 75.0% in patients expressing high levels of menin (*P* = 0.088, Fig. 4b). Finally, lower expression of menin was also associated with a tendency toward worse overall survival (10-year OS of 77.5% versus 85.2%, *P* = 0.092, Fig. 4c).

The abovementioned data obtained in human patients, reminiscent of the observations made in *Men1*-deficient mutant mice, highlight a relationship between reduced menin expression and weaker ERα expression, suggesting that decreased ERα expression triggered by *Men1* deficiency could be related to the occurrence of luminal B-like and ER-negative breast cancer subtypes.

### Menin downregulation alters GATA3 and FOXA1 expression in ER+ breast cancer cells

**Fig. 5:**
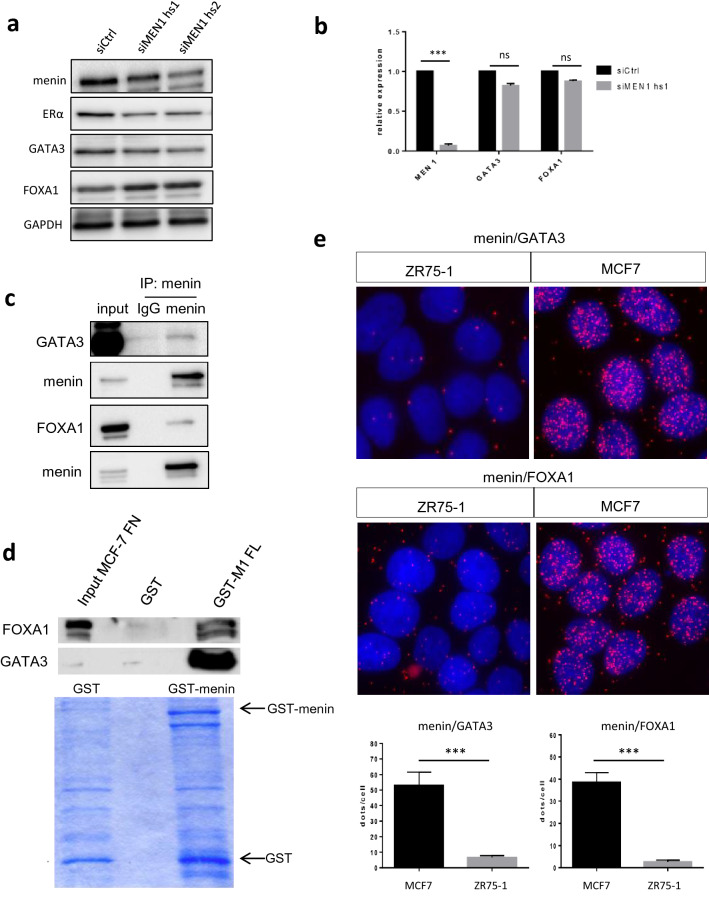
Menin interacts with GATA3 and FOXA1 and influences their expression. **a** Western blot analyses using antibodies against menin, GATA3, and FOXA1 in MCF7 cells treated with siCtrl or siMEN1 hs1. **b** Quantitative RT-qPCR analyses of the *GATA3* and *FOXA1* transcription in MCF7 cells treated with siCtrl or siMEN1 hs1. **c** Co-immunoprecipitation analyses were carried out by incubating nuclear lysates of MCF7 cells with either anti-IgG, or anti-GATA3 or FOXA1 antibodies and subjected to western blot analyses. **d** GST pull-down using GST-full-length (FL) menin and nuclear fraction of protein lysates of MCF7 cells, detected by western blot using the anti-GATA3 or FOXA1 antibodies. Coomassie blue-stained gel showing levels of recombinant GST proteins used in GST pull-down assay. **e** Upper: PLA analysis with anti-menin and anti-GATA3 antibodies in MCF7 and ZR75-1 cells, the latter expressing no menin. Under: the quantification of PLA analysis. All the data are expressed as mean ± SEM, ns *P* > 0.05, *P < 0.05, **P < 0.01, ***P < 0.001

Having demonstrated a clinical correlation between menin inactivation and breast cancer subtypes, we wondered whether the factors important for luminal cell differentiation could be affected by menin in ER+ breast cancer cells. GATA3 is known to be a major factor involved in the regulation of *ESR1* expression and is ubiquitously present in luminal A breast cancers [25]. Western blot analysis revealed that GATA3 expression was greatly reduced in both MCF7 and T47D cells at the protein level after *MEN1* KD, although its level of mRNA was not impacted (Figs. 5a, b, S2). In parallel, we investigated the expression of FOXA1, which plays an important role in mammary cell differentiation and tumorigenesis, and found that its protein expression increased upon *MEN1* KD in MCF7 cells, but remained unchanged in T47D cells, whereas no transcriptional alteration could be detected in both cell lines (Figs. 5a, b, S2). Since GATA3 has been reported to interact with menin in lymphocytes [26], and menin is known to interact with one member of the FOXA family, FOXA2 [27], we performed immunoprecipitation (IP) and PLA analyses to determine whether menin could interact with GATA3 and FOXA1 in breast cancer cells. The data obtained demonstrated that they interact with menin in MCF7 cells, as evidenced by IP at the endogenous level (Fig. 5c), by GST pull-down (Fig. 5d) and by PLA (Fig. 5e). Taken together, the current work revealed that menin interacts both with GATA3 and FOXA1 in ER + breast cancer cells. Moreover, its expression could be critically related to the expression of GATA3, a well-recognized marker of the luminal A subtype.

## Discussion

The current work provides both clinical and experimental data showing that menin is critically involved in ERα expression and that its inactivation in mammary cells is correlated with the occurrence of luminal B and ER-negative breast cancer subtypes. Our data highlighted cellular and molecular consequences of reduced menin expression in mammary cells, which may affect not only cell proliferation but also other hallmarks of cancer cells, in particular, cell differentiation.

We previously observed that the mammary lesions developing in mammary cell-specific *Men1* mutant mice displayed low ERα expression [19]. Our current study further demonstrates that the decrease occurs in the precancerous lesions, suggesting that menin inactivation favors the tumorigenesis of mammary cells with weak ERα expression. Interestingly, by analyzing this expression in a large cohort of breast cancer patients, we found that reduced menin expression is significantly correlated with both ERα-negative and luminal B-like breast cancer subtypes. Consistently, low levels of menin were correlated with larger tumors, more advanced SBR grades, and worse prognosis, all of which are major features of these two breast cancer subtypes [4]. It is worth mentioning that these clinical data, together with those concerning reduced ERα expression in *Men1*-deficient mouse MIC lesions, further support the oncosuppressive role played by the *MEN1* gene in the tumorigenesis of normal mammary gland cells [19, 23]. Moreover, while searching for luminal cell factors likely interacting with menin, we unveiled that menin binds physically to GATA3 and FOXA1 in mammary cells, and that *MEN1* silencing reduces GATA3 expression in MCF7 and T47D cells. Of note, reduced GATA3 expression is often seen in the luminal B breast cancer subtype but not in luminal A [28]. However, the mechanisms leading to the occurrence of both luminal breast cancer subtypes remain elusive. The current work may provide useful insight and generate interest for further studies. In the meantime, considering the retrospective nature of the study and the heterogeneity of the therapies received by the patients included, the clinical analyses, which could be limited with the cutoff definition by IHC, should be confirmed in other cohorts, preferably through prospective studies.

Dreijerink et al. first described the capacity of menin to regulate *ESR1* transcription by binding to the remote upstream part of regulatory sequences of *ESR1*, through an enhancer-mediated looping mechanism, involving GATA3 [23]. Moreover, the occupancy of this enhancer sequence by GATA3 has been reported to play an important role in the regulation of ERα expression upon estradiol stimulation [25]. Our findings provide complementary information related to the role of menin in *ESR1* regulation through its proximal promoter. Intriguingly, our data showed retarded H3K4me3 methylation on the proximal *ESR1* promoter with MI503 treatment, as well as a lack of clear *ESR1* transcriptional alteration after single MLL1, MLL2, or their combined knockdown with siRNA. Since MI503 has been demonstrated not only to inhibit the interaction between menin and MLL1/MLL2 but also to reduce menin expression itself [29]; our data may suggest that factors other than the MLL complex may also participate in this regulation. It would be interesting in the future to identify the factors or cofactors that interact, positively or negatively, with menin to regulate this gene. In addition, our data seem to support the oncogenic role played by menin in ERα+ breast cancer cell lines, the proliferation of which is highly ERα-dependent. Therefore, by combining the data obtained from our experimental and clinical analyses, we consider that menin most likely acts as an oncogenic cofactor in the luminal A breast cancer subtype.

## Conclusion

The emerging role for the *MEN1* gene in mammary cell tumorigenesis appears to be multifaceted. Our current results provide further data showing that menin may play different, even opposite, roles in the development of different breast cancers, in agreement with the findings reported by Dreijerink et al. Taken together, these results may explain seemingly controversial data reported so far, in particular when comparing data obtained from naturally occurring tumors and those of cultured cancer cells. Furthermore, our findings may also raise awareness to the breast cancer subtypes selected when designing new therapeutic strategies involving the eventual use of menin and MLL inhibitors.

## Materials and methods

### Patients

We screened a total of 433 consecutive female patients with breast cancer who underwent surgery and (neo)/adjuvant therapy at the Centre Léon Bérard (CLB) between January 2001 and December 2003 (Additional file 1). Patients with complete data and with adequate samples assessable for menin by IHC were 354, among which 294 patients had ER+/HER2− tumors. The intrinsic subtypes of breast cancer were defined by the histological grade and IHC surrogates as per St Gallen 2013 consensus [30]. Patients were defined as luminal A-like if positive for ER and PR, negative for HER2 expression, and low proliferation (grade I or grade II with low Ki67 or mitotic index). Luminal B-like was defined as ER-positive and either: PR negativity, HER2 positivity or high proliferation. The study was conducted in accordance with the guidelines in the Declaration of Helsinki and the use of all patient tissues was approved by local IRB and carried out according to French laws and regulations.

### TMA analysis of human breast cancers

Formalin-fixed paraffin-embedded breast cancers were prepared and processed for immunostaining as previously described [19]. Tissue micro-array (TMA) block preparation, menin nuclear expression assessment using IHC, and statistical analyses were performed as previously described [19]. The percentage of stained cells was multiplied by the intensity of staining to obtain the H score [31]. For the sake of correlations and survival analyses, the most discriminative cutoff in terms of DFS (as determined by Kaplan–Meier method) was chosen to divide the whole cohort of patients into high menin expression (*H* score > 100) and low menin expression (*H* score ≤ 100).

### Animal breeding

*Men1*^*F/F*^*-WapCre*^+^ and *Men1*^+*/*+^*-WapCre*^+^ mice previously generated in our lab were used [19]. All animal experiments were conducted in accordance with accepted standards of animal care and were approved by the Animal Care and Use Committee of the University Lyon 1.

### Cell culture, transfections, and luciferase assays

Three breast cancer cell lines expressing ERα, namely MCF7, T47D, and ZR75-1, were used in this study. Transient transfections were carried out in phenol red-free medium supplemented with 10% charcoal-stripped serum (Biowest) in order to remove steroid hormones (steroid depletion). Cells were transfected with 20 nM siRNA of, respectively, control siRNA (5 nmoles, Eurogentec), two different siRNA targeting human *MEN1* transcript (siMEN1 hs1 (HSS106462) and hs2 (HSS181079), ThermoFisher Sci.), siRNA targeting human *MLL1* (SiKMT2A: siRNA 107,890 ThermoFisher Sci.), siRNA targeting human *MLL2* (SiKMT2B*:* siRNA s18833 ThermoFisher Sci.) using Jetprime® transfection reagent (Polyplus) for 72 h according to the manufacturer’s instructions. Inhibition of the menin–MLL interaction was achieved by MI503 (Active Biochem) at different concentrations. Prior to performing treatment with E2 and MI503, cells were grown in phenol red-free medium supplemented with 10% charcoal-stripped serum (Biowest) in order to remove steroid hormones (steroid depletion). Cells were then treated for 3 h with E_2_ (Sigma) 10^−8^ M and MI503 for 48 h. The treatment was repeated after 24 h due to the degradation of the inhibitor with time. Please also see Additional file 2—Supplemental Materials & Methods.

### Foci formation assay

For foci formation assay, cells were seeded in 6-well culture plates at 5 × 10^2^ cells for MCF7 and T47D. Cells were transfected with siRNA or treated with MI503 and cultured for 2 weeks. The ensuing colonies were stained with 0.05% crystal violet. The images of the plates were analyzed using ImageJ software. Each experiment was conducted in triplicate and statistical analyses were performed using the Prism software.

### Construction of luciferase constructs

We used genomic DNA extracts from MCF7 cells to generate regions of the human *ESR1* promoter, PrAB (genomic location: Chr6q25.1; 152127793-152129027) and PrC (genomic location: Chr6q25.1; 152124474-152127509) (Additional file 2). The resulting fragments of the proximal *ESR1* promoter were cloned into the pGL3 Basic vector (Promega, Madison, WI).

### Luciferase assays

For luciferase assays, MCF7 cells were cultured in 24-well plate. 48 h after transfection with 250 ng of the reporter plasmid PrAB or PrC, and 5 ng pRL-TK internal control vector, cell lysates were prepared and analyzed using a dual-luciferase reporter assay system (Promega, Madison, WI), as previously reported [27]. Comparisons between mean values were assessed using the two-tailed Student *t* test.

### Real-time reverse transcription and qPCR analyses

RNAs were extracted using RNeasy-Kits (Qiagen, Valencia, USA). Real-Time PCR analyses were carried out on a Step-One RT-System (Applied Biosystem, France) using SYBR-Green (Life Technologies, France) and corresponding primers (Additional file 2). Results of each sample were normalized.

### Protein extraction, immunoprecipitation, GST pull-down, and immunoblotting

Total protein extracts from cells and immunoprecipitation were prepared and analyzed as described previously [27]. For GST pull-down assays, 1.25 µg purified GST menin protein or GST control protein was incubated with 1 mg or 2 mg of nuclear cell extracts prepared from MCF7 cells, as previously described [27]. The co-sedimented proteins were detected by western blot using standard conditions.

### Immunostaining

Tissue preparation, immunostaining, and statistical analyses were performed as previously described [19]. Briefly, endogenous peroxidases were quenched in 3% H_2_O_2_ solution for 30 min at room temperature. Heat-induced epitope retrieval was performed by immersion in antigen-unmasking solution (catalog no. H-3300; Vector Laboratories) in a microwave oven for 15 min. After blocking with antibody diluent (Dako), sections were incubated overnight with a primary antibody (Additional file 2). For immunofluorescence (IF) staining, signals were detected with a Cy3 or Cy5 tyramide amplification kit (PerkinElmer), with prior incubation with the appropriate biotinylated secondary antibody according to the manufacturer’s instructions. Images were acquired on an Eclipse-NiE NIKON microscope using the NIS-Elements Software.

### Proximity ligation assay (PLA), image acquisition, and analysis

MCF7 cells were fixed in methanol for 5 min and washed twice in PBS and then treated and analyzed according to the manufacturer’s instructions (Duolink II Fluorescence, Olink Bioscience, Sweden). Images were acquired on an Eclipse NiE NIKON microscope using the NIS-Elements Software. For each sample, at least one hundred cells were counted. Analysis and quantification of these samples were performed using the ImageJ software (free access). PLA dots were quantified on 8-bit images using the ‘Analyse Particles’ command, while cells were counted using the cell counter plugin.

### ChIP-qPCR assay

Chromatin for ChIP analysis was prepared from 5 million MCF7 or T47D cells. Briefly, cells were fixed in 1% formaldehyde for 10 min, nuclei were obtained and lysed in 300 μl ice-cold RIPA buffer prior to Chromatin-DNA shearing with a Diogene Bioruptor. ChIP was performed using 5 μg of primary antibodies. Dynabeads® Protein G (10003D, Life Technologies, France) was used to retrieve immunocomplexes according to manufacturers’ instructions.

### Statistical analyses

For molecular biology experiments, statistical analyses were performed as described in the Fig. legends; unpaired Student’s *t* tests were used unless otherwise indicated. All analyses were conducted using the Prism5 software (GraphPad, USA); a *P* value of < 0.05 was considered to be significant. Results are expressed as means ± standard errors of the means (SEM). For the patient samples, numerical variables were compared using Student’s *t* test, while categorical variables were compared using *χ*^2^ test. Distant metastasis-free survival (DMFS) was defined as time from diagnosis to the date of distant metastasis or death or last follow-up. Disease-free survival (DFS), defined as the time from diagnosis to death or progression or to date of last follow-up (for censored patients), was also calculated. Survival rates were estimated using the Kaplan–Meier method, and comparisons between menin expression groups were performed using the log-rank test. All statistical tests were two-sided, and the *P* value was considered statistically significant if lower than 5%. Statistical analyses were performed using SPSS 20.0 statistics package.

## Supplementary Information

Below is the link to the electronic supplementary material.

Supplementary file1 (DOCX 16 kb)

Supplementary file2 (DOCX 17 kb)

Supplementary file3 (DOCX 15 kb)

Supplementary file4 (PPTX 6200 kb)

## Data Availability

The data that support the findings of this study are available from the corresponding author, [CXZ for basic research and IT for clinical data analyses], upon reasonable request.
