# Supplementary material for: Reduced menin expression leads to decreased ERα expression and is correlated with the occurrence of human luminal B-like and ER-negative breast cancer subtypes
Source: Breast Cancer Res Treat. 2021 Sep 24;190(3):389–401. doi: 10.1007/s10549-021-06339-9 (PMC8558183; doi:10.1007/s10549-021-06339-9)
Supplement: Supplementary file 1 — Supplementary file1 (DOCX 16 kb) [file 10549_2021_6339_MOESM1_ESM.docx]

| Characteristic | | Number | percent |
| --- | --- | --- | --- |
| Age group | *< 50 years*  *>50 years* | 113  320 | 26.1%  73.9% |
| Menopausal status | *Premenopausal*  *Post-menopausal*  *Unknown* | 121  303  9 | 28.5%  71.5% |
| BMI | *< 25 kg/m^2^*  *> 25 Kg/m^2^* | 258  159  16 | 61.9%  38.1% |
| Tumor size | *<2cm*  *>2cm* | 252  181 | 58.2%  41.8% |
| Axillary LN metastasis | *No*  *Yes* | 184  249 | 42.5%  57.5% |
| SBR grade | *I*  *II*  *III* | 82  207  144 | 18.9%  47.8%  33.3% |
| ER status | *Negative*  *Positive* | 56  377 | 12.9%  87.1% |
| PR status | *Negative*  *Positive* | 109  324 | 25.2%  74.8% |
| HER2 status | *Negative*  *Positive*  *Missing* | 397  31  5 | 92.8%  7.2% |
| Breast cancer subtype | *Luminal A*  *Luminal B*  *HER2 enriched*  *TNBC* | 243  134  11  45 | 56.1%  30.9%  4.6%  10.4% |
| Adjuvant hormonal regimen | *Tamoxifen*  *Tam-AI*  *Missing* | 173  198  62 | 46.6%  53.4% |

**Supplemental Table 1** Clinico-pathological characteristics of the tested patient cohort (433 patients)
