# Supplementary material for: Reduced menin expression leads to decreased ERα expression and is correlated with the occurrence of human luminal B-like and ER-negative breast cancer subtypes
Source: Breast Cancer Res Treat. 2021 Sep 24;190(3):389–401. doi: 10.1007/s10549-021-06339-9 (PMC8558183; doi:10.1007/s10549-021-06339-9)
Supplement: Supplementary file 2 — Supplementary file2 (DOCX 17 kb) [file 10549_2021_6339_MOESM2_ESM.docx]

***Supplemental materials and methods***

*Cell culture conditions*

MCF7 and T47D cells were cultured in DMEM (Gibco Invitrogen) containing 25 mM glucose supplemented with 10% (v/v) foetal calf serum (FCS), 2 mM L-Glutamine, 100 unit/ml Penicillin, 100 μg/ml streptomycin, MEM non-essential amino acids solution (1X), 100 mM β-Mercaptoethanol, at 37°C with 5% CO_2_. T47D cells cultured in RPMI medium (Gibco Invitrogen) containing 25 mM glucose and supplemented with 10% (v/v) foetal calf serum (FCS), 2 mM L-Glutamine, 100 unit/ml Penicillin, 100 μg/ml streptomycin, HEPES 10 mM, Sodium Pyruvate 1 mM, at 37°C with 5% CO_2_.

*Primers used in the study*

Primers for qRT-PCR:

*MEN1 Fw: 5’-CATCCAGGACTACAACTACT-3’ ;*

*MEN1 Rev: 5’-ATGACATCATTGGCTACTTC-3’;*

*ESR1 Fw: 5’- GGCTGCAAGGCCTTCTTCAA-3’;*

*ESR1 Rev: 5’-CCTGGCAGCTCTTCCTCCTG-3’;*

*TFF1 (Ps2) Fw: 5’-TTGTGGTTTTCCTGGTGTCA -3’;*

*TFF1 (Ps2) Rev: 5’-CCGAGCTCTGGGACTAATCA -3’;*

*GATA3 Fw: 5’-CAGACCACCACAACCACACTCT-3’;*

*GATA3 Rev* : 5’-GGATGCCTTCCTTCTTCATAGTCA-3’

Primers used for ChIP:

*ESR1 -2500*. Fw: 5’-GGAGCACCTCAGTATGCGT -3’;

Rev: 5’-CTCTCATTTCCCAGTGGCGT-3’;

*ESR1 -2000* Fw: 5’-TTTCCTCTTGCTTGGGGTGG-3’;

Rev 5’-TACTGGGGAGGAGAAACCCC-3’;

*ESR1 -1400*  Fw: 5’-GCCCTATGAAGTGCTTTTTGCAT-3’;

Rev: 5’-ACACATTAGGTGCATCGCAG-3’;

*ESR1* -800 Fw: 5’-GCCCAGTGAACCGAGAAGA-3’;

Rev: 5’-CGGTCTGGTCCAGCTAACAG-3’;

*ESR1 +1 TSS* Fw: 5’-TATCCAGCAGCGACGACGACCAG-3’;

Rev: 5’-ATCACTCCAGGCACAACTCG-3’;

*ERS1* + 400 Fw: 5’-CATCTGGGATGGCCCTACTG-3’;

Rev: 5’-CTCGGGGTAGTTGTACAGG-3’;

*ESR1*  +1200 Fw: 5’-CAGGCAAATAAACACGGGGC-3’;

Rev: 5’-GCTAACTCCAAACACCCCCA-3’;

*ESR1* +2000 Fw: 5’-GCCTGCAGAGGGATTAGGTG-3’;

Rev: 5’-ATTCAGGACCCAGCCATGCG-3’;

*Chr1 Neg primers*. Fw: 5’-CGGGGGTCTTTTTGGACCTT-3’;

Rev: 5’-GAAACACGGCTGCCAGAAAC-3’.

Primers for Luciferase constructs:

PrAB motif the forward primer was 5′-GATGCTAGCTATCCTAGCCCAAGTGAACCG-3’ and the reverse primer was 5′-GCAGATCTGTGCAGACCGTGTCCCCGCAG-3’.

For PrC motif the forward primer was 5’-GAAGGTACCAGCATTTGGGCTGGACCTATT-3’ and the reverse primer was 5’-CATAAGCTTATCTGACGAAGTGTTCATGGTCT-3’.

*The primary antibodies used in the study*

For western blot : rabbit anti-menin at 1/8000 (Bethyl laboratories, TX, USA), rabbit anti-ERα 1/4000 (Bethyl laboratories), rabbit anti-GATA3 (Bethyl laboratories), mouse anti-actin monoclonal antibody from ICN (1:50 000, Aurora, CA, USA). For GST: with primary antibodies goat mouse anti-GATA3 at 1/2000 (Santa Cruz Bio, CA, USA). For immunostaining: goat anti-menin at 1/8000 (Bethyl laboratories), rabbit anti-ER 1/4000 (Santa Cruz Biotechnologie). For PLA, rabbit anti-GATA3 1/2000 (Bethyl laboratories) and goat anti-menin 1/2000 (Bethyl laboratories). For ChIP, anti-menin antibody (A300-105A, Bethyl Laboratories Inc.), anti-H3K4me3 (C15410003, Diagenode, Belgium).

*ChIP-qPCR assay*

DNA recovered was then extracted with phenol/chlorophorm/isoamylalcoohol, following proteinase-K reverse-crosslinking. 2 μl of IP-DNA or 2 ng of INPUT DNA were used for Real Time PCR analysis to quantify co-precipitated chromatin-DNA. Relative enrichment of a given promoter region obtained with a specific antibody was compared with input DNA. Blocks of the region on Chr1 was used as a negative control for menin and H3K4me3 ChIPs.
