# Supplementary material for: Reduced menin expression leads to decreased ERα expression and is correlated with the occurrence of human luminal B-like and ER-negative breast cancer subtypes
Source: Breast Cancer Res Treat. 2021 Sep 24;190(3):389–401. doi: 10.1007/s10549-021-06339-9 (PMC8558183; doi:10.1007/s10549-021-06339-9)
Supplement: Supplementary file 3 — Supplementary file3 (DOCX 15 kb) [file 10549_2021_6339_MOESM3_ESM.docx]

***Legends for Supplemental Figures***

**Fig. S1 Alteration of H3K4me3 makers on the proximal *ESR1* promoter in *MEN1*-KD MCF7 cells.** ChIP-qPCR analysis with anti-H3K4me3 antibody to the -2500 bp / +2000 bp area flanking the transcription start site (TSS) on *ESR1* in MCF7 cells treated or not with MI503 at a concentration of 2 µM, and tested at 72 h (upper panel) and 96 h (lower panel) after the treatment.

**Fig. S2 Menin and the expression of GATA3 and FOXA1 in T47D cells.** quantitative RT-qPCR (**a**) and Western blot (**b**) analyses detecting GATA3 and FOXA1 expression in T47D cells treated with siCtrl or siMEN1 hs1.
