# Supplementary material for: Reduced menin expression leads to decreased ERα expression and is correlated with the occurrence of human luminal B-like and ER-negative breast cancer subtypes
Source: Breast Cancer Res Treat. 2021 Sep 24;190(3):389–401. doi: 10.1007/s10549-021-06339-9 (PMC8558183; doi:10.1007/s10549-021-06339-9)
Supplement: Supplementary file 4 — Supplementary file4 (PPTX 6200 kb) [file 10549_2021_6339_MOESM4_ESM.pptx]

## Slide 1
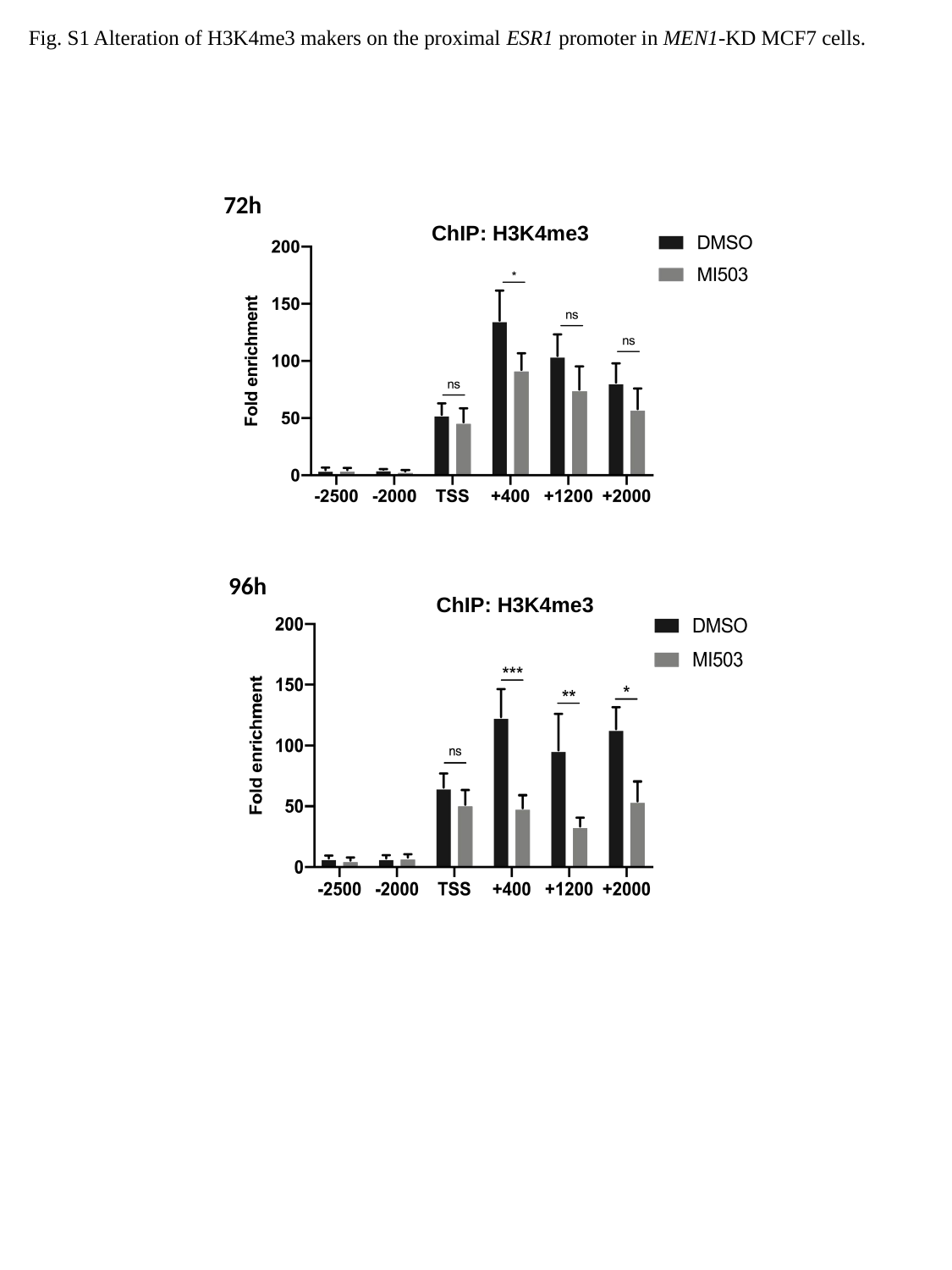

Fig. S1 Alteration of H3K4me3 makers on the proximal ESR1 promoter in MEN1-KD MCF7 cells.
72h
ChIP: H3K4me3
96h
ChIP: H3K4me3

## Slide 2
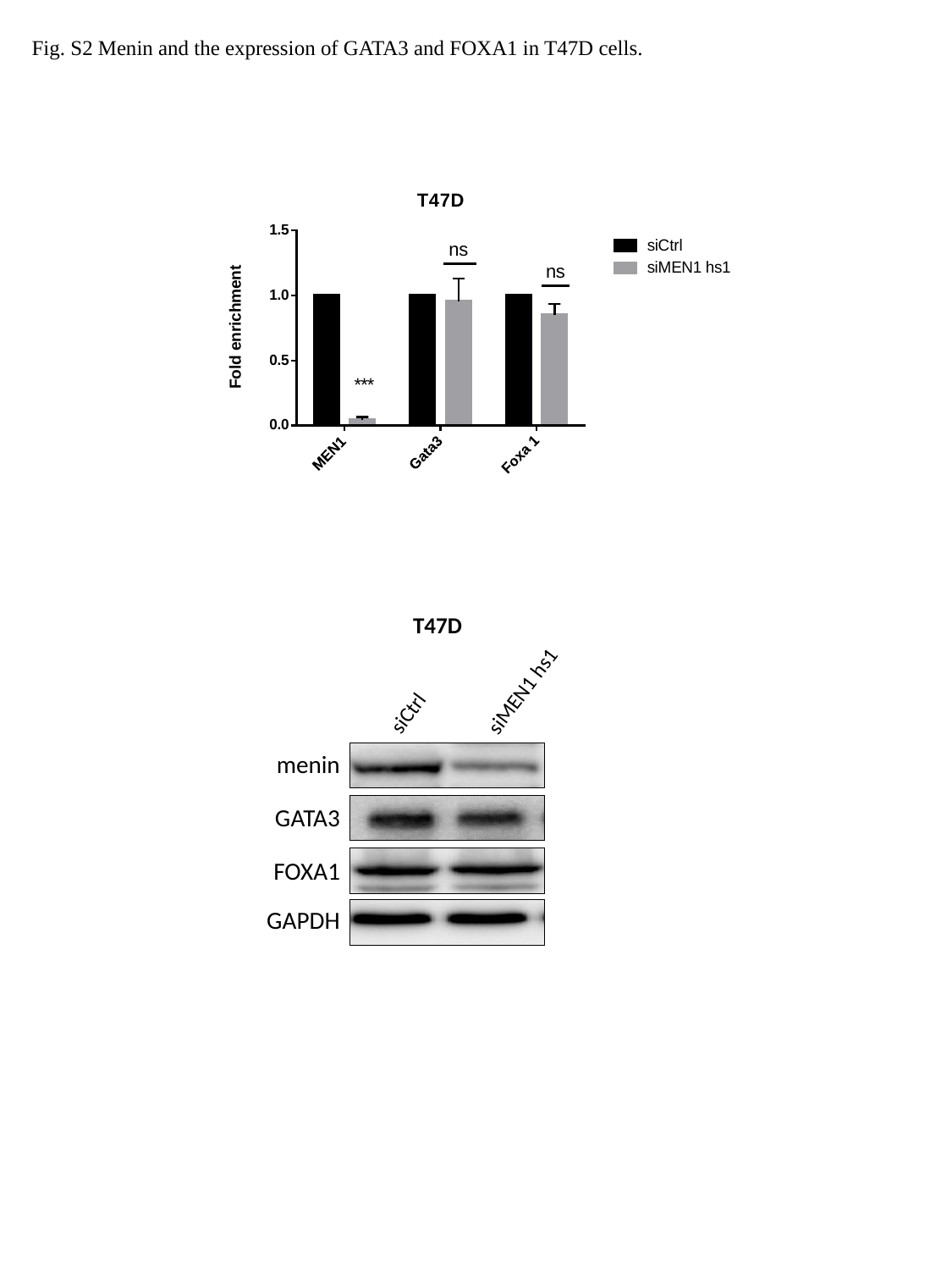

Fig. S2 Menin and the expression of GATA3 and FOXA1 in T47D cells.
T47D
siMEN1 hs1
siCtrl
menin
GATA3
FOXA1
GAPDH
